# Cannabinoids in Chronic Pain: Clinical Outcomes, Adverse Effects and Legal Challenges

**DOI:** 10.3390/neurolint17090141

**Published:** 2025-09-05

**Authors:** Aleksandar Sic, Conor George, Daniela Ferrer Gonzalez, Vasilis-Spyridon Tseriotis, Nebojsa Nick Knezevic

**Affiliations:** 1 Department of Anesthesiology, Advocate Illinois Masonic Medical Center, Chicago, IL 60657, USA; aca.smed01@gmail.com (A.S.); conor.george@my.rfums.org (C.G.); daniferrer1990@gmail.com (D.F.G.); 2 Chicago Medical School, Rosalind Franklin University of Medicine and Science, North Chicago, IL 60064, USA; 3 Department of Neurology, Agios Pavlos General Hospital of Thessaloniki, Leoforos Ethnikis Antistaseos 161, Kalamaria, 55134 Thessaloniki, Greece; vasilistseriotis@hotmail.com; 4 Laboratory of Clinical Pharmacology, Aristotle University Campus, Aristotle University of Thessaloniki, 4124 Thessaloniki, Greece; 5 Department of Anesthesiology, University of Illinois, Chicago, IL 60612, USA; 6 Department of Surgery, University of Illinois, Chicago, IL 60612, USA

**Keywords:** endocannabinoid system, pain management, analgesia, cannabinergic therapy, chronic pain management, cannabis-based medicine

## Abstract

Cannabinoids have gained increasing attention as potential therapeutic agents in chronic pain management. Their mechanisms of action, mediated through CB1 and CB2 receptors, provide a pharmacological alternative to conventional analgesics. The evidence is strongest for neuropathic pain and multiple sclerosis-related spasticity, while the results for fibromyalgia, osteoarthritis, and musculoskeletal pain remain inconsistent. The average pain reduction is modest, often not exceeding 0.5–1.0 points on a 10-point scale, and therapeutic gains are offset by safety concerns. Quantitative data show that discontinuation rates range from 4.3% at low-dose CBD to 12.9% at high-dose CBD, compared with 3.5% on placebo, while nabiximols (THC + CBD spray) are associated with dizziness in 25% of patients, somnolence in 8%, and treatment discontinuation in 12%. High-dose CBD also carries a measurable risk of hepatotoxicity. Regulatory heterogeneity further constrains trial feasibility, scalability, and patient access, with disparities evident across the United States, Europe, Canada, and Australia. Overall, cannabinoids provide modest, condition-specific analgesia and should be considered adjunctive rather than first-line options, reserved for patients unresponsive to conventional therapy. Future progress requires standardized formulations, harmonized international regulations, long-term safety data, and large-scale randomized controlled trials to clarify their role in evidence-based pain management.

## 1. Introduction

Chronic pain is a debilitating condition experienced by approximately one in five adults worldwide. It impacts around 1.5 billion people and increases in prevalence with age [1]. In the United States alone, an estimated 20.9% of adults, or roughly 51.6 million people, suffer from chronic pain, while 6.9% (17.1 million) live with high-impact chronic pain that significantly limits daily activities [1]. The financial burden is substantial: annual costs in the United States range from USD 560 to 635 billion, including healthcare expenditures and lost productivity [2]. Globally, the impact is similarly profound, with chronic pain ranked among the top ten causes of years lived with disability (YLDs), accounting for substantial productivity loss and impaired daily functioning [3].

Standard treatments for chronic pain include nonsteroidal anti-inflammatory drugs (NSAIDs), anticonvulsants, antidepressants, physical therapy, and opioids. While these therapies can provide relief, each class carries important safety concerns, including gastrointestinal, cardiovascular, or central nervous system adverse effects [3,4]. For instance, in acute lower back pain, NSAIDs demonstrate a number needed to treat (NNT) of 14 for significant pain reduction, 12 for reduced disability, and 13 for global improvement within three weeks [5]. In chronic musculoskeletal pain, topical NSAIDs like diclofenac gel have an NNT of approximately 4.6 for pain improvement over two weeks [6]. Antidepressants like duloxetine show benefit in neuropathic conditions, with an NNT of six for diabetic peripheral neuropathy and eight for fibromyalgia for ≥50% pain relief [7]. In addition to pharmacological strategies, clinical guidelines emphasize non-pharmacological interventions, including structured exercise programs, cognitive behavioral therapy, and multidisciplinary rehabilitation, which improve function and quality of life in patients with chronic pain [8]. International guidelines further frame the management of chronic pain. The American College of Physicians (ACP) recommends exercise therapy, mindfulness-based stress reduction, and cognitive behavioral therapy as first-line treatments, while reserving pharmacological options, including NSAIDs and duloxetine, for cases where non-pharmacological strategies prove insufficient [8]. The International Association for the Study of Pain (IASP) promotes a multimodal and interdisciplinary model of care. It does not endorse cannabinoids as first-line therapy but identifies them as an area requiring further research and clinical evaluation [9].

Approximately 22% of adults with chronic pain in the United States reported using prescription opioids in the past three months, with usage being highest among adults aged 45 to 64 years (25.9%) and lowest among young adults aged 18 to 29 years (11.8%) [10]. These data highlight the continued reliance on pharmacologic therapies even though comparative analyses demonstrate moderate efficacy at best and a higher risk profile than recommended non-pharmacological interventions. Long-term opioid use is further complicated by tolerance, dependence, and life-threatening adverse events and has played an important role in the opioid crisis [11]. Against this backdrop, interest has grown in exploring alternative or adjunctive approaches to chronic pain management. One area of particular attention is the endocannabinoid system, through which endocannabinoids and phytocannabinoids like Δ^9^-tetrahydrocannabinol (THC) and cannabidiol (CBD) modulate nociceptive signaling and inflammatory responses, producing potential analgesic and anti-inflammatory effects in conditions including neuropathy, multiple sclerosis, arthritis, and musculoskeletal disorders [12]. Epidemiological studies indicate that approximately 10–15% of patients with chronic pain use cannabis for symptom management [13]. In patients with musculoskeletal pain, 23% reported cannabis use, and more than 60% of these individuals rated it as effective, with many reporting reduced reliance on opioids or other analgesics [13]. Despite these encouraging observations, the evidence base remains limited by heterogeneity in product formulations, dosing protocols, and study designs, as well as by restrictive regulatory frameworks that continue to limit the feasibility of large-scale, long-term clinical trials.

Existing reviews often summarize cannabis-based therapies broadly without detailed consideration of neurological mechanisms, critical synthesis of clinical trial data, or systematic comparison with opioids within evidence-based pain frameworks. Evidence on long-term safety and regulatory integration also remains fragmented. Therefore, this review aims to (a) synthesize current evidence on cannabis for chronic pain, with emphasis on neurological pathways of analgesia; (b) critically evaluate clinical trial data on efficacy and safety; (c) compare cannabinoids with opioids and conventional therapies; and (d) identify gaps in the knowledge and regulatory barriers that must be addressed to guide future research and clinical implementation.

## 2. Historical Perspectives on Medical Cannabis

The origins of medicinal cannabis are uncertain. Numerous ancient civilizations documented the use of herbal remedies to alleviate pain, promote sedation, reduce inflammation, and address emotional distress. Historical texts from ancient Egypt, China, the Assyrian Empire, Greek mythology, and the Roman Empire all reference the use of plant-based vapors or infusions for these purposes [14].

In the early 1800s, Europe rediscovered the medicinal and psychoactive properties of cannabis through the translation of Arabic texts and manuscripts by scholars and physicians during the era of European colonial expansion into India and the Middle East [14]. However, cannabis was not integrated into Western medicine until the late 19th century.

In 1878, J. Russell Reynolds, Physician-in-Ordinary to Queen Victoria, published over 30 years of clinical experience with cannabis in The Lancet. The main challenge that remained was the inability to identify active compounds, making dosage and therapeutic standardization difficult due to variability in cannabis strains, cultivation, and origin [14]. Despite these limitations, Reynolds reported the efficacy of cannabis in treating facial neuralgia, migraine, dysmenorrhea, and paresthesia associated with gout, among other conditions. By the end of the 19th century, over 100 scholarly articles on medicinal cannabis had been published in Europe and the United States [15]. However, progress was paused in the 1940s due to legal restrictions that significantly restricted research efforts.

The growing medical use of cannabis was reduced by U.S. political developments and racialized anti-cannabis propaganda, which portrayed it as a drug of abuse and threatened the hemp industry. This culminated in the passage of the 1937 Marihuana Tax Act, which imposed strict penalties on both medical and industrial cannabis use, despite opposition from the American Medical Association [16].

## 3. Endocannabinoid System and Analgesic Mechanisms

The endocannabinoid system (ECS) is a complex lipid signaling network that plays a critical role in maintaining physiological homeostasis and modulating pain sensation, inflammation, appetite, mood, and neuroprotection [17]. It comprises three main components: receptors, endogenous ligands, and metabolic enzymes. G protein-coupled receptors—mainly CB1 and CB2—mediate most of the known effects of cannabinoids and represent the primary receptor classes within the system.

### 3.1. Cannabinoid Receptors (CB1, CB2, TRPV1, and PPAR)

**Figure 1:**
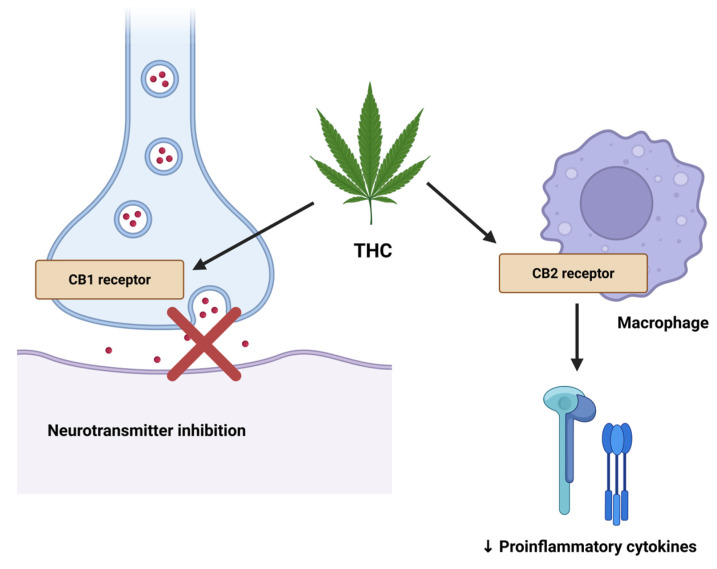
Cannabinoid receptor-mediated mechanisms of action. Δ^9^-tetrahydrocannabinol (THC) binds to CB1 receptors on presynaptic neurons, resulting in inhibition of excitatory neurotransmitter release, and to CB2 receptors on macrophages, leading to reduced production of proinflammatory cytokines. Created in BioRender online application, available at https://app.biorender.com/ (accessed on 18 July 2025).

CB1 receptors are predominantly found in the central nervous system and modulate neurotransmitter release, while CB2 receptors are mainly located on immune cells and play a role in modulating inflammation. Cannabinoids bind to CB1 receptors on presynaptic neurons and inhibit the release of excitatory neurotransmitters (glutamate and substance P), whereas CB2 receptor activation reduces the production of proinflammatory cytokines [18] (Figure 1). Transient receptor potential vanilloid type 1 (TRPV1) channels respond to physical stimuli, chemical agents, and specific ions and contribute to ECS signaling. Peroxisome proliferator-activated receptors (PPARs) also regulate ECS activity through their nuclear receptor function.

### 3.2. Endogenous Ligands and Enzymatic Regulation (AEA, 2-AG, FAAH, and MAGL)

**Figure 2:**
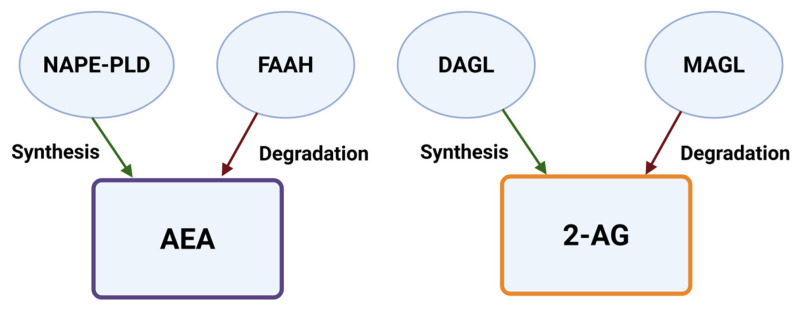
Synthesis and degradation of endogenous ligands. Anandamide (AEA) is generated by NAPE-PLD and degraded by FAAH, while 2-AG is synthesized by diacylglycerol lipases (DAGL-α/β) and degraded by monoacylglycerol lipase (MAGL). Created in BioRender online application, available at https://app.biorender.com/ (accessed on 28 August 2025).

Anandamide (AEA) and 2-arachidonoylglycerol (2-AG) are the main endogenous ligands and bind to ECS receptors under physiological conditions [19,20]. Diacylglycerol lipase isozymes alpha and beta, fatty acid amide hydrolase (FAAH), monoacylglycerol lipase (MAGL), and N-acylphosphatidylethanolamine-specific phospholipase D regulate the synthesis and degradation of these ligands [21] (Figure 2). The physiological importance of the ECS has prompted the development of therapies that target its components by modulating receptor activity or inhibiting endocannabinoid metabolism.

### 3.3. Central vs. Peripheral Mechanisms of Pain Modulation

Peripherally, CB1 receptors on nociceptor terminals reduce membrane excitability and suppress the release of glutamate and substance P, while CB2 receptors on macrophages and other immune cells limit cytokine release and inflammatory sensitization in injured tissue and dorsal root ganglia. Proof-of-concept work with peripherally restricted CB1 and CB2 agonists shows attenuation of peripheral afferent sensitization without central side effects, underscoring the therapeutic appeal of targeting the periphery [22].

**Figure 3:**
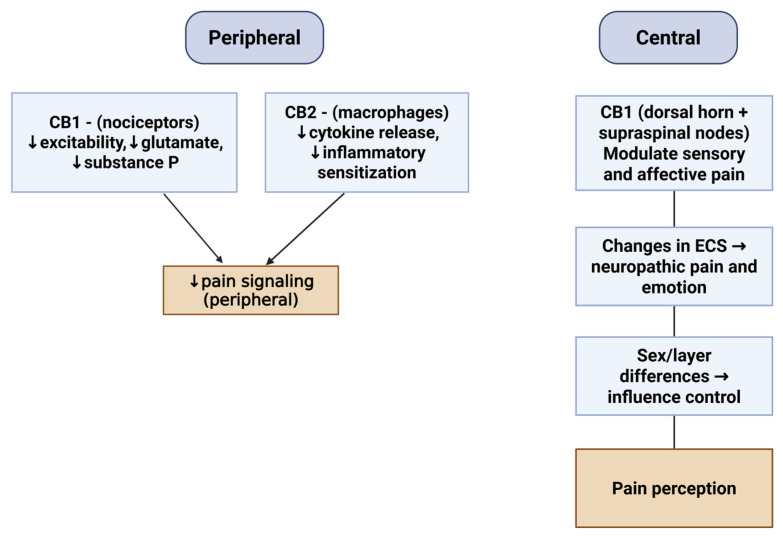
Central and peripheral mechanisms of pain modulation mediated by the endocannabinoid system. Created in BioRender online application, available at https://app.biorender.com/ (accessed on 28 August 2025).

Centrally, CB1 is enriched in the superficial dorsal horn and in supraspinal nodes that shape both sensory and affective pain dimensions, including the periaqueductal gray, thalamus, and amygdala. Differences in CB1 expression between sexes and across spinal layers also influence how pain signals are controlled [23]. These mechanisms are summarized in Figure 3.

Neuroimmune crosstalk contributes at both sites. Targeting CB2 modulates neurogenic inflammation in migraine and other conditions and provides a non-neuronal entry point for analgesia [24].

### 3.4. Synaptic Plasticity and Maladaptive Changes

Endocannabinoids are retrograde messengers that depress transmitter release through presynaptic CB1, producing short-term depression and long-term depression at excitatory and inhibitory synapses. These forms of plasticity, classically observed as depolarization-induced suppression of inhibition or excitation, are prominent in spinal and cortical pain circuits [25].

Postsynaptic eCB release requires synucleins and a SNARE-dependent mechanism, providing a molecular handle on how activity drives eCB-mediated suppression in pain-relevant networks. Nanoscale presynaptic organization also tunes tonic cannabinoid signaling and synapse-specific variability, which may influence susceptibility to central sensitization [26,27].

**Figure 4:**
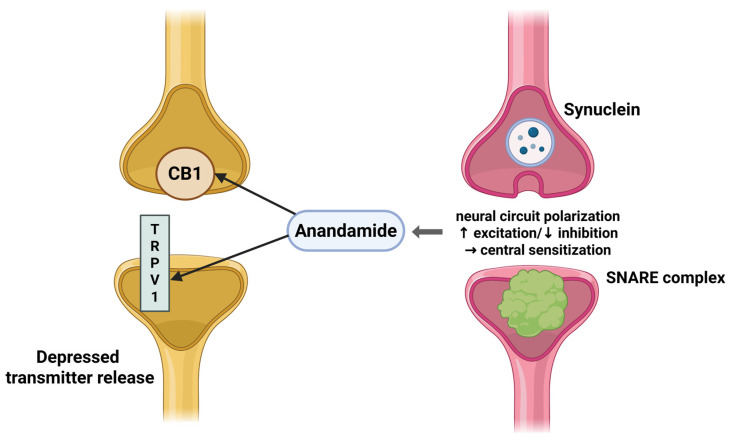
Postsynaptic anandamide release modulates presynaptic CB1/TRPV1, leading to depressed transmitter release and neural circuit polarization underlying central sensitization. Created in BioRender online application, available at https://app.biorender.com/ (accessed on 28 August 2025).

At the spinal level, anandamide acting via CB1 and TRPV1 shapes dorsal horn transmission, and neural circuit polarization describes a shift toward heightened excitation and weakened inhibition in chronic pain [28,29] (Figure 4).

### 3.5. Glial Modulation and Neuroinflammation

Microglia and astrocytes are active drivers of central sensitization. CB2 signaling dampens microglial activation, reduces proliferation, and lowers release of TNF-α and IL-1β. CB2-directed strategies normalize microglial reactivity and microglia–astrocyte crosstalk, with benefits for network function and behavior in neuroinflammatory models [30,31].

**Figure 5:**
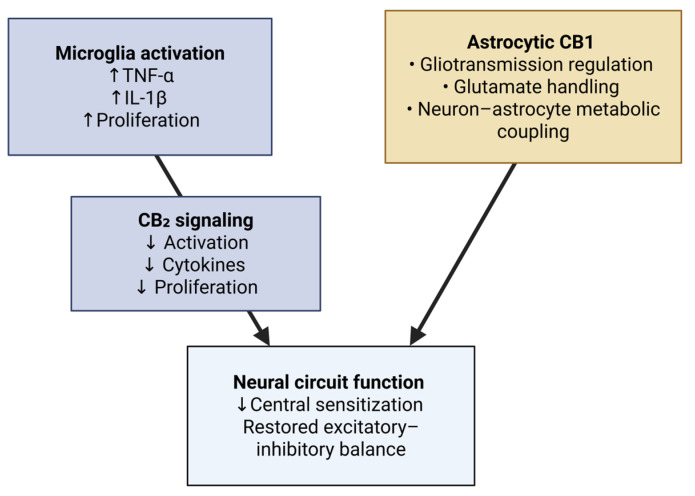
Glial modulation and neuroinflammation. CB_2_ signaling dampens microglial activation and cytokine release, while astrocytic CB1 regulates gliotransmission and glutamate handling. Together with peripheral CB_2_ effects, these mechanisms restore excitatory–inhibitory balance and reduce central sensitization. Created in BioRender online application, available at https://app.biorender.com/ (accessed on 28 August 2025).

Astrocytic CB1 regulates gliotransmission, glutamate handling, and metabolic coupling with neurons. Astrocytes also modulate baseline neurotransmission and eCB-dependent plasticity, positioning astroglial ECS as a lever to restore excitatory–inhibitory balance in chronic pain states. Peripheral CB2 mechanisms in arthritic joints additionally illustrate how local immune modulation feeds back on central gain [32] (Figure 5).

### 3.6. Descending Pain Modulation

**Figure 6:**
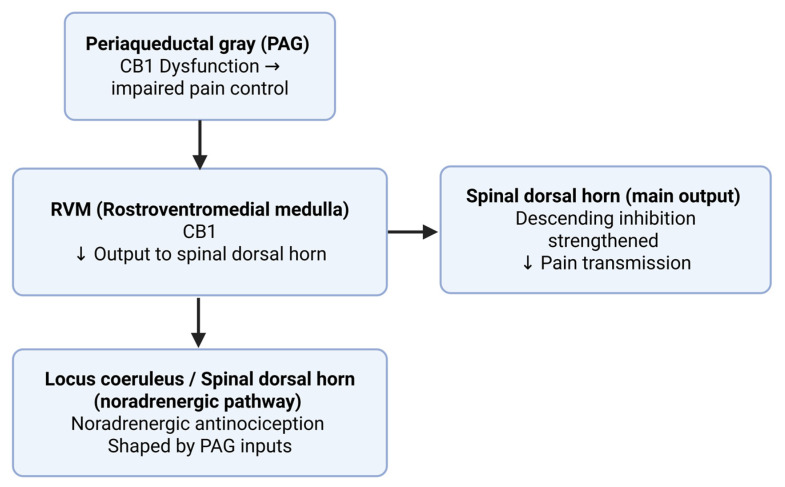
Cannabinoid modulation of the descending pain pathway. CB1 receptors in the periaqueductal gray (PAG) and rostroventromedial medulla (RVM) regulate output to the spinal dorsal horn, while PAG inputs to the locus coeruleus shape noradrenergic antinociception. These circuits enhance descending inhibition and reduce pain transmission. Created in BioRender online application, available at https://app.biorender.com/ (accessed on 28 August 2025).

Cannabinoids engage the descending pain modulatory system. CB1 within the periaqueductal gray and rostroventromedial medulla adjusts output to the spinal dorsal horn and interacts with serotonergic and noradrenergic pathways to strengthen descending inhibition. Periaqueductal gray dysfunction is associated with impaired endogenous pain control in chronic lower back pain, consistent with the role of PAG–RVM circuits as a central component of cannabinoid analgesia [33]. Inputs from the periaqueductal gray to the locus coeruleus shape noradrenergic antinociception, and GPR55 may also contribute within the PAG–RVM–spinal axis, broadening cannabinoid-related targets beyond CB1 and CB2 [34] (Figure 6).

## 4. Cannabinoids in Pain Management

Cannabinoids can provide modest but measurable relief in chronic pain, although variability in study design continues to limit interpretation. The strongest data come from neuropathic pain, where cannabinoids reduced pain scores by 6 to 9 points on a 0 to 100 scale and nearly doubled the likelihood of achieving a 30% reduction [35]. Patients who used cannabis also reduced opioid consumption by about 64% [36]. This suggests a role for cannabinoids in conditions where conventional therapies provide insufficient benefit, yet the diversity of doses, delivery routes, and endpoints makes it difficult to generalize results.

This pattern extends to multiple sclerosis, where oromucosal nabiximols reduced pain and spasticity by about 1 point on a 0 to 10 scale [37,38]. The improvements are clinically meaningful for patients unresponsive to agents like baclofen or tizanidine, but the overall effects remain modest. Outcomes varied, with some studies emphasizing pain and others spasticity or global function, which further complicates interpretation. While the gains in MS appear incremental, the results for migraine highlight a more rapid and pronounced effect. Inhaled cannabis containing 6% THC and 11% CBD relieved pain in 67.2% of patients and eliminated pain completely in 34.5% within two hours, significantly surpassing the placebo, with effects lasting up to 48 h [39,40,41]. These results point to cannabinoids as potentially fast-acting options, although inconsistent endpoints make it challenging to compare efficacy directly with triptans or NSAIDs.

Across all indications, cannabinoids produced only small reductions in pain intensity, typically 4 to 9 points on a 0 to 100 scale [35]. Thus, while cannabinoids can relieve pain at a level comparable to standard pharmacological options in some patients, their modest effect size, frequent adverse events, and highly variable dosing strategies show the need for standardized formulations and longer comparative trials before their role in chronic pain management can be firmly established.

## 5. Ongoing Trials and Emerging Therapeutic Data

Recent trials broaden our understanding of cannabinoids in chronic pain, yet they consistently reveal how fragile and context-dependent the evidence remains. In fibromyalgia, oral THC-rich oil titrated to around 30 mg per day improved fatigue, daily function, and overall symptom burden, whereas inhaled THC/CBD altered pressure pain thresholds without reducing spontaneous pain [42,43]. The discrepancy between oral and inhaled preparations underscores how the route of administration and achievable dosing can determine whether effects translate into meaningful clinical benefits.

When researchers moved to systemic conditions, the results became even less convincing. In sickle cell disease, vaporized cannabis did not reduce pain intensity and produced only minor improvements in mood [44]. Cannabidivarin for HIV-associated neuropathic pain likewise failed to improve symptoms or quality of life [45]. These results point to a limited role of cannabinoids in pain states, driven by widespread systemic or inflammatory mechanisms, in contrast with localized or neuropathic conditions where targeted delivery may matter more.

Evidence from osteoarthritis illustrates this divergence. Topical CBD gel applied to the thumb joint reduced pain and improved hand function without safety issues [46], while a novel metered inhaler delivering very low doses of THC relieved neuropathic and complex regional pain without cognitive impairment [47]. In stark contrast, high-dose oral CBD at 600 mg daily in knee osteoarthritis offered no analgesic benefit and instead caused elevated liver enzymes [48]. Efficacy ultimately depends on the type of cannabinoid, the formulation, the dose, and the site of action, with evidence showing that higher doses do not necessarily provide stronger or safer analgesia.

A structured overview of these and other recent randomized controlled trials investigating cannabinoids in various chronic pain conditions is presented in Table 1.

In parallel with completed trials, several ongoing clinical studies are actively investigating how different cannabinoid compounds, formulations, and treatment strategies may alleviate chronic pain. These trials vary widely in design, reflecting the complexity of cannabinoid pharmacology and the need to tailor interventions to specific pain mechanisms.

One phase 2 study is evaluating low-dose aerosolized delta-9-tetrahydrocannabinol (Δ^9^-THC) delivered via a precise metered-dose inhaler. This formulation enables controlled pulmonary absorption and aims to reduce peripheral neuropathic pain in patients with diabetes. The trial is testing whether targeted THC inhalation can provide effective analgesia without significant psychoactive or systemic side effects [49].

A separate investigation is focusing on oral synthetic cannabidiol (CBD), testing two fixed doses in patients recovering from orthopedic trauma. The primary goal is not to treat established chronic pain but to determine whether early intervention with CBD can prevent the onset of persistent pain following acute injury. This reflects a shift toward preventive pain management using non-opioid strategies [50].

The role of cannabinoids in central pain processing is being examined in a mechanistic trial using vaporized cannabis containing 5% THC. This study is exploring how THC influences pain perception and neural activity in both healthy individuals and those with ongoing pain. It integrates neuroimaging and behavioral data to clarify the pathways through which cannabis exerts its effects [51].

In a large-scale open-label trial, researchers are studying inhaled medical cannabis across a wide range of chronic pain conditions, including neuropathic pain, cancer-related pain, and PTSD. This trial uses a digitally enabled inhaler that adjusts strain and dosage depending on the clinical indication. Unlike traditional randomized trials, this pragmatic design seeks to generate real-world data on effectiveness and tolerability in a diverse patient population [52].

Dronabinol, a synthetic version of THC formulated in oral capsules, is being tested in women with chronic pelvic pain due to endometriosis. This trial targets pain that has been unresponsive to hormonal and conventional therapies, aiming to assess whether pure THC can offer symptomatic relief in hormonally driven, inflammation-related pain syndromes [53].

Oral CBD is also under evaluation in a large population of individuals with chronic musculoskeletal and neuropathic pain. A phase 2 randomized trial is examining whether CBD alone can provide safe and meaningful analgesia, especially in populations where opioid alternatives are urgently needed. Importantly, the study tracks both pain outcomes and adverse events to better understand CBD’s therapeutic window [54].

In a more comparative approach, a multi-arm trial is assessing three distinct cannabinoid treatments: pure THC (Syndros), pure CBD (Epidiolex), and a combined THC–CBD formulation (nabiximols). These are being tested against a placebo in patients with high-impact neuropathic pain. The goal is to identify which formulation, if any, provides the greatest benefit while minimizing side effects. Such direct comparisons are essential to guide rational prescribing decisions [55].

Another ongoing pilot trial is investigating the effects of oral CBD, both alone and in combination with THC oil, in patients with chronic non-palliative pain. This study is particularly focused on optimizing cannabinoid-based interventions within transitional pain care models, where early, balanced analgesia is crucial to avoiding long-term opioid dependence [56].

An overview of these ongoing clinical trials, including key details on study design, target populations, and cannabinoid formulations, is summarized in Table 2.

All these completed and ongoing studies are showing the therapeutic potential and complexity of using cannabinoids for treating chronic pain. While some formulations appear beneficial in specific pain syndromes (fibromyalgia and localized osteoarthritis), others show minimal efficacy or raise safety concerns. The diversity in study populations, cannabinoid types, dosages, and delivery methods reflects the evolving nature of this research field.

## 6. Regulatory Barriers and Patient Access

Although patient interest in cannabinoid-based therapies for chronic pain continues to grow, and several studies have demonstrated moderate efficacy, clinical implementation remains limited due to inconsistent regulations and significant geographic variability.

### 6.1. United States and North America

In the United States, cannabis remains classified as a Schedule I substance at the federal level, which restricts clinical research and complicates funding. This classification has resulted in a fragmented system in which individual states establish their own rules. Currently, 38 states and the District of Columbia have approved medical cannabis programs, but access remains highly uneven, influenced by socioeconomic status, racial disparities, and local regulatory frameworks [57,58]. For example, a recent study from Pennsylvania (2018–2024) reported a fourfold difference in medical cannabis certification rates for pain between counties, with higher uptake observed in more affluent, predominantly white areas [59].

In Canada, medical cannabis has been legal since 2001, yet access varies across provinces. In early 2025, Health Canada enacted amendments streamlining the Cannabis Regulations, easing licensing requirements for applicants and licensees, reducing administrative burden, and facilitating medicinal research and operational efficiencies. At the same time, a 2025 proposal introduced a new regulatory pathway for CBD products, aiming to reclassify certain CBD items under the Natural Health Products (NHPs) category, with requirements for strict labeling, licensing, and a THC cap of 0.001% [60,61].

### 6.2. Europe and International Perspectives

In Europe, Germany and the United Kingdom allow physicians to prescribe standardized cannabinoid formulations, including nabiximols. However, reimbursement remains restricted, and there are significant gaps in clinical evidence supporting their effectiveness in chronic pain management. Nabiximols received full regulatory approval in the United Kingdom in 2010 and in Germany in 2011. Despite this, access remains hindered by funding challenges, particularly within systems such as the NHS, which have been reluctant to cover associated costs [62]. Meta-analyses of 32 to 36 randomized controlled trials with follow-up periods of up to two weeks indicate small to moderate reductions in pain in patients with non-cancer chronic pain, along with modest opioid-sparing effects. However, data on long-term safety, dosing standardization, and direct comparisons with other treatments are still lacking [63]. Recent regulatory changes highlight how approaches differ between countries. In Germany, the Cannabis Act of 2024 legalized possession of small amounts of cannabis, permitted home cultivation of up to three plants, and allowed adults to join non-profit social clubs for personal use. Medical cannabis continues to be regulated under the existing framework, while commercial sales remain prohibited [64]. In Switzerland, physicians have been authorized to prescribe cannabis without special approval since 2022 [65].

### 6.3. Global Guidelines and Future Directions

Globally, regulatory guidance remains inconsistent. The U.S. Food and Drug Administration (FDA) has not approved the cannabis plant or whole-plant extracts for chronic pain, authorizing only specific cannabinoid-based medicines such as Epidiolex (CBD), Marinol, and Syndros (dronabinol) for other indications [66]. In 2024, the Drug Enforcement Administration (DEA) proposed rescheduling delta-9 THC from Schedule I to Schedule III, a change that could ease barriers to research and pharmaceutical development [67]. Best practice guidelines from the American College of Physicians advise against inhaled cannabis and recommend cautious use of oral formulations, particularly avoiding use in adolescents, pregnant women, and individuals with psychiatric or substance use disorders [68]. International clinical guidelines propose starting with low-dose CBD, gradually titrating upward, and introducing THC only when necessary and under strict supervision [69,70].

Abroad, regulatory positions remain similarly conservative but are beginning to evolve. In the United Kingdom, NICE continues to recommend against the routine use of cannabis for chronic pain outside of clinical trials, citing insufficient long-term evidence [71]. In Australia, the Therapeutic Goods Administration has acknowledged that medicinal cannabis provides clinically significant pain relief only in a minority of patients, with a number needed to treat of about 22 for a 30% reduction in pain and 26 for a 50% reduction. The agency, therefore, recommends cautious, case-by-case consideration, particularly after standard therapies have failed [72]. Updated guidance issued in late 2024 and early 2025 recommends a “start low, go slow” approach and highlights the importance of shared decision making between patients and clinicians. At the same time, regulatory scrutiny has intensified: nearly half of the evaluated prescribing clinics were found to be in breach of TGA standards, and the Australian Health Practitioner Regulation Agency (AHPRA) has taken disciplinary action against dozens of practitioners for unsafe prescribing and misleading practices [72].

Overall, despite incremental progress, global perspectives on medical cannabis remain fragmented. Most authorities continue to support cautious, restricted use rather than broad adoption. These developments underscore the urgent need for harmonized international standards, stronger long-term evidence, and equitable access frameworks to establish cannabinoids as a safe and effective option in chronic pain management.

## 7. Adverse Effects, Safety Concerns, and Misuse Potential

Cannabinoid therapies in chronic pain provide only modest analgesic benefits while exposing patients to frequent and sometimes disabling adverse effects. The reported outcomes indicate mild to moderate disturbances of the central nervous and gastrointestinal systems, but incidence rates and long-term safety remain poorly documented [73]. High-THC-to-CBD formulations reduce pain intensity by approximately 0.5 to 1.0 points, yet the supporting evidence is weak, and adverse event frequencies remain incompletely characterized [68]. Clinical data consistently reveal dizziness, dry mouth, fatigue, nausea, headache, somnolence, confusion, disorientation, and vision disturbances as the most prevalent adverse outcomes [74,75]. Rates of dizziness reach 25%, drowsiness 8%, and disorientation 4%, with up to 12% of patients discontinuing treatment due to side effects [76,77]. Serious adverse events vary by indication, reaching 21.8% in cancer pain compared with 16.9% for placebo, 4.7% versus 0.8% in multiple sclerosis spasticity, and 4.1% versus 3.1% in neuropathic pain [68]. In real-world practice, discontinuation occurs in 6.3% of patients within twelve weeks, most commonly due to dizziness, confusion, fatigue, or throat irritation [74].

Population-specific risks remain insufficiently studied. Individuals with cannabis use disorder experience nearly a threefold increase in all-cause mortality and approximately a tenfold higher suicide risk over five years. THC exposure induces acute anxiety, cognitive impairment, psychotic-like symptoms, and higher accident risk, while chronic use contributes to persistent attention and memory deficits, especially with adolescent onset [78,79,80]. CBD poses distinct risks, as hepatotoxicity occurs in 5.6% of healthy adults at moderate doses, a finding absent in placebo groups, underscoring clinically relevant liver injury potential [81]. CBD also alters serum levels of medications such as warfarin and tacrolimus through inhibition of CYP2C9, CYP3A4, and related metabolic pathways [82]. Modern cannabis products contain dramatically higher levels of THC than in the past, with the average potency rising from about 4–5% in the 1990s to 15–20% today [83,84]. This escalation in psychoactive strength enhances the drug’s addictive potential, as higher THC exposure accelerates dependence and increases the likelihood of cannabis use disorder [85]. Neuroimaging and neurochemical studies confirm that high-THC cannabis stimulates dopaminergic activity in the substantia nigra and ventral tegmental area, regions directly implicated in psychosis. By amplifying dopamine release, modern cannabis formulations create a biological bridge between recreational use and the emergence of psychotic symptoms [86]. Daily recreational use of high-potency cannabis increases the odds of developing a psychotic disorder by nearly five times compared with non-users, while regular daily use in general triples that risk (adjusted odds ratio ≈ 4.8 for high-potency; ≈ 3.2 for daily use) [87,88]. These magnitudes are clinically relevant, placing cannabis alongside well-established risk factors for psychosis rather than as a minor or incidental contributor. Across 11 European sites, daily cannabis use accounted for about 20.4% of new psychosis cases, while daily use of high-potency cannabis explained approximately 12.2% [87]. In Amsterdam, daily cannabis use may contribute to 43.8% of first-episode psychosis cases and high-potency cannabis to 50.3%; in London, these figures are 21.0% and 30.3%, respectively [87,89,90]. These city-level differences show how variations in cannabis availability and potency can directly shape local incidence of psychotic disorders, emphasizing that epidemiological patterns are not uniform but influenced by policy and market dynamics. Furthermore, about 0.5% (or 1 in 200) of cannabis-exposed individuals experience a full cannabis-associated psychotic episode, while 19–21% experience more transient psychotic-like symptoms, such as paranoia, hallucinations, or delusions [88]. Even these so-called transient reactions may carry functional consequences, since repeated exposure increases the likelihood of persistence and escalation. Among those who experience cannabis-induced psychosis, around 34% will transition to a schizophrenia diagnosis, a higher proportion than transitions linked to hallucinogens (26%) or amphetamines (22%) [88]. This progression shows that cannabis-induced psychosis is not simply an acute toxic state but a potential entry point into a chronic psychiatric trajectory, particularly in young and vulnerable populations.

This divergence between standardized medical products and unregulated recreational cannabis further complicates the evidence base. Standardized pharmaceutical preparations like nabiximols provide greater dose control and quality assurance, whereas recreational products often deliver uncontrolled THC concentrations of uncertain purity. Evidence supporting cannabis in chronic pain remains inconsistent, and authoritative guidelines report no demonstrable efficacy in work-related chronic pain conditions [79,91]. Overall, cannabinoids offer analgesic promise, but the current literature lacks accurate adverse event quantification, dose–response characterization, and differentiation between THC and CBD effects. Vulnerable groups, including psychiatric patients, older adults, and individuals with substance use disorders, are underrepresented, highlighting the need for stratified and rigorously controlled studies to achieve a balanced risk–benefit assessment.

## 8. Discussion and Limitations

The current evidence shows that cannabinoids have a limited but measurable role in chronic pain therapy. The strongest support comes from neuropathic pain and multiple sclerosis-related spasticity, where patients consistently report pain relief, functional improvement, and better quality of life [92,93,94,95]. The results for fibromyalgia, osteoarthritis, and musculoskeletal pain are inconsistent, which indicates that cannabinoids should serve as a secondary option when standard treatments fail [96,97,98]. Average pain reduction with cannabinoids rarely exceeds 0.5 to 1.0 points on a 10-point scale, which further raises concerns about clinical relevance. Duloxetine produces significantly larger reductions [99,100]. Gabapentin provides meaningful relief for approximately 35% of patients, compared with 21% with placebo [101]. The evidence does not confirm a superior short-term efficacy of opioids in neuropathic pain, which suggests a weaker comparative benefit overall [102,103].

These benefits are consistently offset by safety concerns. Patients commonly experience dizziness, fatigue, cognitive slowing, anxiety, and psychiatric symptoms, and discontinuation rates are considerable [74,75,76,77,104,105]. Meta-analytic data confirm that in oral CBD trials, discontinuation reached 4.3% at low doses (20–400 mg), 8.8% at medium doses (600–1000 mg), and 12.9% at high doses (1400–3000 mg), compared with 3.5% with placebo (OR = 2.61; 95% CI: 1.38–4.96). In studies of nabiximols (THC + CBD spray), dizziness occurred in 25%, somnolence in 8%, and disorientation in 4% of patients, while 12% discontinued treatment due to adverse events [106,107,108]. High-dose CBD is also linked to hepatotoxicity [81,82]. Beyond trial settings, individuals with cannabis use disorder show higher mortality and elevated suicide risk [78,79,80]. All of these results are showing the need for strict patient selection and continuous monitoring, while the lack of standardized adverse event reporting continues to limit reliable assessment [92,93].

Methodological limitations further weaken the evidence base. Trials vary in THC-to-CBD ratios, dosing regimens, routes of administration, and outcomes, which inflates pooled estimates and complicates comparability [93,103]. Many studies enroll a high proportion of prevalent cannabis users, which limits generalizability to cannabis-naïve patients, while small sample sizes and DerSimonian–Laird models produce overly narrow confidence intervals and misleading impressions of precision [109,110]. Short follow-up periods, usually less than one year, further preclude firm conclusions on long-term safety, dependence, and cumulative toxicity [78,93,103], and rare but severe events often remain underpowered to detect [97,111].

Socio-legal factors add another layer of complexity. Restrictive legislation hampers high-quality trial design, while commercial promotion and online advocacy amplify expected benefits and minimize risks, creating unrealistic patient expectations and complicating evidence-based counseling [112]. In the United States, cannabis remains a Schedule I substance, a designation that restricts federal funding, complicates trial approval, and restricts standardized formulation development [113]. Recently introduced legislation, such as the Evidence-Based Drug Policy Act of 2025, aims to ease these restrictions [114], while the DEA’s potential move to reschedule cannabis to Schedule III could facilitate research access and reduce regulatory burdens [115]. Regulatory heterogeneity persists globally. Some European countries permit prescribing (for example, Germany and the Netherlands), whereas others maintain restrictive regimes that hinder multinational trial harmonization [116]. This diversity limits patient access and research scalability [117]. Consequently, legal frameworks not only determine trial feasibility and external validity but also shape the accessibility of cannabinoid-based therapies in clinical practice.

In conclusion, cannabinoids provide modest, condition-specific analgesia but remain unsuitable as a universal therapy for chronic pain. Their role is adjunctive, best reserved for carefully selected patients with neuropathic pain or multiple sclerosis-related spasticity where conventional therapy has failed. At present, adverse effects, safety uncertainties, methodological weaknesses, and socio-legal restraints highly outweigh the possible benefits.

## 9. Conclusions

Cannabinoid-based therapies represent a promising yet still evolving option for chronic pain management. Their mechanisms, primarily mediated through CB1 and CB2 receptor pathways, offer a novel pharmacological approach distinct from conventional analgesics. Current clinical evidence supports modest, condition-specific efficacy, particularly in neuropathic pain and multiple sclerosis–related spasticity, while results in fibromyalgia and localized osteoarthritis remain inconsistent. However, therapeutic gains are offset by frequent adverse effects, with up to one quarter of patients experiencing dizziness, sedation, or cognitive impairment, and discontinuation rates being nearly three times higher than with placebo. High-dose CBD also poses hepatotoxic risks, highlighting the importance of dose optimization and safety monitoring. Beyond clinical data, regulatory inconsistencies continue to limit both research scalability and patient access. The potential rescheduling of cannabis in the United States and ongoing reforms in Europe, Canada, and Australia illustrate how legal frameworks directly influence trial feasibility, physician confidence, and equitable availability.

Overall, cannabinoids should be considered adjunctive rather than first-line therapy, reserved for carefully selected patients who do not achieve sufficient relief with standard treatments. Future progress requires large-scale randomized controlled trials, pragmatic real-world studies, standardized formulations, long-term safety evaluations, and harmonized international regulations to define the precise role of cannabinoids in evidence-based pain care.

## Figures and Tables

**Table 1:** Summary of recent randomized controlled trials (RCTs) on cannabinoids for chronic pain. THC—Δ^9^-tetrahydrocannabinol; CBD—cannabidiol; CBDV—cannabidivarin; SCD—sickle cell disease; FIQ—Fibromyalgia Impact Questionnaire; VAS—visual analog scale; DASH—Disabilities of the Arm, Shoulder, and Hand questionnaire; OA—osteoarthritis; QoL—quality of life; AE—adverse event; RCT—randomized controlled trial; TKA—total knee arthroplasty.

Study (Author, Year)	Population	Intervention	Control	Outcome	Conclusion
van de Donk et al., 2019 [42]	20 patients with fibromyalgia (chronic muscle pain)	Single inhalation of pharmaceutical cannabis: 3 strains (THC-dominant, CBD-dominant, and THC + CBD combination)	Inhaled placebo (without THC/CBD)	No difference in spontaneous pain between cannabis and placebo. However, the THC + CBD combination showed a higher proportion of patients achieving ≥30% pain reduction (90% vs. 55%; *p* = 0.01). THC-containing strains increased pressure pain threshold (*p* < 0.01). No serious adverse effects.	Limited short-term analgesic effect of THC (with/without CBD) in fibromyalgia.
Chaves et al., 2020 [43]	17 women with fibromyalgia (low socioeconomic status)	Oral THC-rich cannabis oil (24 mg/mL THC; 0.5 mg/mL CBD); titrated to ~30 mg THC/day for 8 weeks	Oral placebo oil	Significant reduction in Fibromyalgia Impact Questionnaire (FIQ) scores in THC group compared with placebo (*p* = 0.005). Improvements noted in well-being, pain intensity, work ability, and fatigue. No severe adverse effects.	THC oil improved fibromyalgia symptoms and was well tolerated.
Abrams et al., 2020 [44]	23 adults with sickle cell disease (SCD) and chronic pain (crossover study)	Inhaled cannabis (vaporizer) with 4.4% THC and 4.9% CBD, 3×/day for 5 days (in-hospital)	Inhaled placebo (no active components)	No significant difference in daily average pain ratings. Only minor mood-related improvements (*p* = 0.02). No effect on sleep, physical activity, or opioid use. Well tolerated.	Cannabis not superior to placebo for pain relief in SCD.
Eibach et al., 2021 [45]	32 patients with HIV-related neuropathic pain (crossover RCT)	Oral cannabidivarin (CBDV), 400 mg/day for 4 weeks	Oral placebo (identical format)	No reduction in neuropathic pain with CBDV vs. placebo (pain score slightly worse with CBDV; *p* = 0.16). No impact on analgesic use, pain features, or quality of life. Well tolerated.	CBDV was safe but ineffective for HIV-related neuropathic pain.
Heineman et al., 2022 [46]	18 patients with base-of-thumb osteoarthritis (hand joint pain; crossover)	Topical CBD gel (6.2 mg/mL in shea butter), applied 2× daily for 2 weeks	Topical placebo gel (same base)	CBD gel significantly reduced pain compared with placebo (VAS 5.0→2.2 vs. 4.9→4.0). Improved hand function (DASH score). No adverse events or local irritation.	Topical CBD effective for localized joint pain without side effects.
Almog et al., 2020 [47]	27 patients with chronic neuropathic pain or CRPS	Inhaled metered-dose THC (0.5 mg and 1.0 mg) via Syqe Inhaler	Inhaled placebo (identical device)	1.0 mg of THC significantly reduced pain intensity (*p* = 0.014); 0.5 mg showed milder, non-significant effect. No cognitive decline or serious AEs.	Low-dose inhaled THC provided dose-dependent analgesia and was well tolerated.
Pramhas et al., 2023 [48]	86 patients with chronic knee osteoarthritis pain (~63 years old; 8 weeks of treatment)	Oral CBD capsules (600 mg/day) + acetaminophen (3 g/day)	Placebo capsules + same acetaminophen	No additional pain relief with CBD vs. placebo after 8 weeks (WOMAC pain score reduced equally in both groups; *p* = 0.80). Adverse effects more common in CBD group, including elevated liver enzymes.	High-dose CBD not effective and associated with more side effects.

**Table 2:** Summary of ongoing clinical trials investigating the use of cannabinoids for the treatment of chronic pain across various populations and indications. CBD: cannabidiol, a non-psychoactive compound derived from cannabis; THC: delta-9-tetrahydrocannabinol, the primary psychoactive component of cannabis; Δ^9^-THC: full designation of THC (delta-9-tetrahydrocannabinol); CNP: chronic neuropathic pain; PTSD: post-traumatic stress disorder; RCT: randomized controlled trial, a study design that randomly assigns participants to an intervention or control group; VA: Veterans Affairs, referring to U.S. federal healthcare institutions serving military veterans; RYAH inhaler: a smart device for precise dose-controlled delivery of medical cannabis; Syndros: an oral liquid formulation of synthetic THC (dronabinol); Epidiolex: a purified, FDA-approved oral solution of cannabidiol (CBD); Nabiximols: a standardized combination of THC and CBD, commercially known as Sativex^®^.

Study ID	Location(s)	Type of Cannabinoid	Target Population	Status	Expected Completion
NCT06490445 [49]	USA and other countries (multicenter aerosol study)	Aerosolized medical cannabis (~0.25–1.0 mg of Δ^9^-THC per inhalation via Syqe inhaler)	Adults with diabetic peripheral neuropathic pain	Recruiting (phase 2)	Nov 2025
NCT06448923 [50]	Montreal, Canada (trauma center)	Oral CBD (synthetic, two-dose regimen)	Patients with polytrauma (long bone fracture) and acute pain (preventing chronic pain)	Not yet recruiting (phase 2)	Sep 2026
NCT04982965 [51]	United States (UC San Diego and others)	Inhaled (vaporized) cannabis (5% THC by weight) vs. placebo	Adults (21–65; healthy volunteers and chronic pain model)	Recruiting (phase 1)	Mar 2027
NCT03944447 [52]	Multiple U.S. states (multicenter)	Inhaled medical cannabis (doses/strains vary by condition; via RYAH inhaler)	~200,000 patients across ≥33 chronic conditions (including chronic pain, neuropathic pain, cancer pain, PTSD, etc.)	Recruiting (open-label phase 2)	Dec 2025
NCT06834997 [53]	Texas Children’s Hospital (Houston, USA)	Oral dronabinol capsules (synthetic Δ^9^-THC, up to 30 mg/day)	~75 women with endometriosis-related chronic pelvic pain (refractory to standard treatment)	Not yet recruiting (phase 2 pilot RCT)	Dec 2027
NCT06213233 [54]	Michigan and partner VA sites (USA)	Oral CBD solution vs. placebo	468 military veterans with chronic pain (musculoskeletal, neuropathic, etc.)	Enrolling (phase 2 RCT)	Dec 2026
NCT05351801 [55]	VA San Diego (CA), Seattle (WA), and San Antonio (TX), USA	Oral THC, CBD, THC + CBD (4 arms: THC [Syndros], CBD [Epidiolex], THC + CBD [nabiximols] vs. placebo)	Veterans with chronic neuropathic pain (high-impact CNP), ≥21 years	Active, recruiting (phase 2 RCT)	Jun 2027
NCT05351905 [56]	Toronto General Hospital (Ontario, Canada)	Oral CBD (alone) or CBD + THC oil vs. placebo	~51 adults with chronic non-palliative pain (Toronto General Transitional Pain Service)	Recruiting (pilot RCT, phase 2)	Apr 2026
